# miR-93-5p enhance lacrimal gland adenoid cystic carcinoma cell tumorigenesis by targeting BRMS1L

**DOI:** 10.1186/s12935-018-0552-9

**Published:** 2018-05-09

**Authors:** Jie Hao, Xin Jin, Yan Shi, Hong Zhang

**Affiliations:** Department of Ophthalmology, The First Affiliated Hospital of Harbin Medical University, 194 Xuefu Road, Harbin, 150001 Heilongjiang China

**Keywords:** miR-93-5p, Tumorigenesis, BRMS1L, Plasma, Tissue, Adenoid cystic carcinoma, Lacrimal gland

## Abstract

**Background:**

Lacrimal adenoid cystic carcinoma (LACC) is one of the most common malignancies that affects lacrimal gland. MicroRNAs are known to play a crucial role as oncogenes or tumor suppressors. Specifically, miR-93 has been reported to play a crucial role in colorectal, breast, pancreatic, lung cancer and hepatocellular carcinoma. However, the role of miR-93 in LACC and the potential molecular mechanisms involved remain unknown. Therefore, we took the challenge to determine the involvement of miR-93 in the LACC by targeting BRMS1L.

**Method:**

A total of 5 adenoid cystic carcinoma (ACC) of lacrimal gland patient tissues and their plasma were examined. Three normal lacrimal glands and three normal serums were collected as a control group. After surgical resection, the specimens were preserved in liquid nitrogen and stored at − 80 °C until RNA extraction. Afterwards, LACC cells with miR-93-5p overexpression were subjected to qRT-PCR and western blot for epithelial–mesenchymal transition (EMT) markers levels. Ability of LACC cell migration, invasion, proliferation and apoptosis was examined by wounded healing, transwell, CCK-8 and apoptosis assays. Afterwards, TargetScan was used to predict putative targets of miR-93-5p. Then, the examination was performed whether miR-93-5p targets BRMS1L by the use of luciferase reporter assays and western blotting. Finally, immunohistochemical staining was sone and all the images were taken using a microscope (Nikon, Tokyo).

**Results:**

Our results showed that miR-93 was overexpressed in tissues and plasma of LACC patients compared to healthy controls. MiR-93 downregulated E-cadherin expression while increasing N-cadherin expression and significantly inhibited luciferase activity. Furthermore, western blotting results confirmed that miR-93-5p could inhibit BRMS1L expression. The BRMS1L staining in LACC tissues was weaker than in normal controls. In addition, miR-93-5p revealed a reverse correlation with the expression of BRMS1L. In addition, significant upregulation of E-cadherin and downregulation of N-cadherin were found when LACC cells were transfected with BRMS1L. Finally, miR-93-5p significantly enhanced TOP/FOP luciferase activity. Upregulation of BRMS1L reduced TOP/FOP luciferase activity while further overexpression of miR-93-5p could not rescue Wnt signaling activity.

**Conclusions:**

Our findings report that miR-93 promotes LACC cell migration, invasion, and proliferation via targeting downregulation of BRMS1L through regulation of Wnt signaling pathway.

**Electronic supplementary material:**

The online version of this article (10.1186/s12935-018-0552-9) contains supplementary material, which is available to authorized users.

## Background

Although primary lacrimal gland tumors are not prevalent very much (5–25% of orbital lesions) [1] adenoid cystic carcinoma (ACC) has been reported to be the most frequently seen form of malignant epithelial lacrimal gland tumor (66% of malignant lesions) [2–4]. Lacrimal gland adenoid cystic carcinoma (LACC) usually carries a poor prognosis of 5-year survival rate of 50% and 10-year survival rate of only 20% due to osseous and/or perineural invasion with retrograde intracranial extension, and hematogenous or lymphatic spread [1, 5–7]. Clinically, ACC of lacrimal gland is manifested with symptoms such as ocular pain, globe dystopia, proptosis, and ‘S’ shaped ptosis [2]. The main characteristics of LACC are multiple recurrences, intracranial extension, and potential distant metastases to the lung, bone, brain, and liver [8, 9]. The histopathological features of ACC include its heterogeneous morphology in form of tubular, cribriform, and/or solid architectural patterns, that can be also encountered in other salivary gland-type neoplasms [2, 10]. The current treatment protocol includes globe-sparing surgery followed by external radiotherapy (RT), proton-beam therapy, intra-arterial chemotherapy, or radical orbital exenteration [5, 11]. This tumor is rarely present in adults and even less common in children and adolescents, with a mean age of diagnosis of 30–40 years [3, 4, 12, 13].

MicroRNAs (miRNAs) are known to play a crucial role as oncogenes or a tumor suppressors in tumorigenesis, thus being key regulators of tumor progression and metastasis [9, 14]. Those are small, noncoding RNAs of 20–25 nucleotides in length, that regulate posttranscriptional gene expression [15]. However, the literature bearing on the potential role of miRNAs in LACC is scarce. Zhang et al. reported that decreased levels of miR-24-3p function as a tumor suppressor in human LACC by suppressing proliferation and migration/invasion while promoting apoptosis [16].

MiR-93 has been reported to play a crucial role in colorectal, breast, pancreatic, lung cancer and hepatocellular carcinoma [17]. Fang et al. [18] reports that miR-93 promotes tumor growth and angiogenesis. In turn, Fabbri et al. investigated the role of miR-93-5p in the neuroblastoma SK-N-AS cell line [19], while Chen et al. 215 for the first time reported that miR-93-5p hampers epithelial ovarian carcinoma (EOC) tumorigenesis and progression [20]. The role of miR-93-5p–mediated regulation in tumor aggressiveness have a potential to reveal the molecular mechanisms underlying cancer aggressiveness. However, the targetome of miR-93-5p in LACC cell tumorigenesis has not been fully defined so far.

According to Edmonds et al. breast cancer metastasis suppressor 1 (BRMS1) regulates metastasis-associated microRNA expression and metastasis of multiple tumor types without blocking tumorigenesis. BRMS1 enhanced expression of tumorigenesis suppressing miRNAs (miR-146a, -146b and -335), suggesting that miRNA might be involved in the regulation of cancer metastasis [21]. Especially that BRMS1 has been already reported to inhibit breast cancer [22], melanoma [23], non-small cell lung [24] and ovarian cancer metastasis in xenograft and syngeneic models [16].

As microRNA (miRNA) are involved in neoplastic progression, we hypothesized that BRMS1 may also exert some of its metastatic effects by regulating miRNA expression [21]. This is the first study to determine the involvement of miR-93 in the LACC by targeting BRMS1L (Breast Cancer Metastasis-Suppressor 1 Like).

## Materials and methods

### Patients

A total of 5 adenoid cystic carcinoma (ACC) of lacrimal gland patient tissues and their plasma were provided by The First Affiliated Hospital of Harbin Medical University, Heilongjiang, China, from 2011 to 2015. Three normal lacrimal glands and normal serums were collected as control group from patients with eye trauma. No patients received any anticancer treatment before admission. After surgical resection, the specimens were preserved in liquid nitrogen within 5 min of excision and stored at − 80 °C until RNA extraction.

Clinical information was retrieved from the medical history and pathology reports of patients. The clinical data used for qRT-PCR analysis are shown in Table 1. Informed written consent for research purposes was obtained from the patients before tissue collection. The study has been approved by the ethical committee of Harbin Medical University.

**Table1:** Clinical features of patients with adenoid cystic carcinoma of lacrimal gland

Case no.	Sex	Age	Size of tumor
Patient 1	Female	55	2.7 cm × 1.4 cm
Patient 2	Male	40	2.7 cm × 1.7 cm
Patient 3	Male	81	1.8 cm × 2.5 cm
Patient 4	Female	49	2.4 cm × 1.8 cm
Patient 5	Male	29	1.9 cm × 2.0 cm

### RNA extraction and quantitative reverse transcription polymerase chain reaction (qRT-PCR)

Total RNA was extracted from the tissues of 5 samples using TRIzol Reagent (Invitrogen Carlsbad, CA) according to the manufacturer’s instructions. The concentration of RNA was determined using a NanoDrop Spectrophotometer (NanoDrop Technologies, Wilmington, DE). cDNAs were synthesized from total RNA using gene specific primers from cDNA synthesis kit (HIGH-Capacity cDNA Reverse Transcription Kits, USA, TaqMan^®^MicroRNA Reverse Transcription Kits, USA). Quantitative real-time PCR was performed using SYBR Green Real-Time PCR Kit (TaqMan^®^Fast Universal PCR Master Mix, USA) according to the manufacturer’s protocols. U6 and GAPDH were used as internal control. The Ct value is defined as the fractional cycle number at which the fluorescence exceeds the fixed threshold. The fold change was calculated using the 2^−ΔΔCt^ method. All experiments were performed in triplicate. The sequences of each primer were listed in Additional file 1: Table S1.

### Cell isolation and culture

Lacrimal adenoid cystic carcinoma (LACC) stem cell isolation and culture was performed as previously described [25, 26] All cell transfections were performed using X-tremeGENE siRNA Transfection Reagent (Roche, Mannheim, Germany) according to the manufacturer’s protocol.

### Wounded healing assay

LACC cells were seeded into six-well plates and grown to 80–90% confluence. A wound was produced by a straight scratch with a 200 μl sterile pipette tip. The LACC cells were then rinsed with phosphate buffered saline (PBS) to remove the floating cells. Images were captured within 12, 24 h post-wound. The relative distance of cell migration to the scratched area was measured and a healing percentage was calculated. Each test was carried out in triplicate for more than two independent experiments.

### Transwell assay

Transwell chambers containing polycarbonate membrane filters with a 24-well 8-μm pore size (Corning, Corning, NY) were coated with Matrigel. In each well, 40 μl of Matrigel was added to an insert and dried in a 37 °C incubator for 30 min to form a thin gel layer. Then, 24 h after LACC cell transfection, the cells were resuspended with reduced serum Dulbecco’s Modified Eagle Medium: Nutrient Mixture F-12 (DMEM/F-12) and were adjusted to 2.5 × 10^5^ cells/mL. After 24 h, Matrigel and cells remaining on the upper side of the membrane were wiped off, and the cells that had migrated to the bottom surface of the membrane were fixed in 4% paraformaldehyde in PBS. Once fixed, the cells were stained with crystal violet for 10 min at room temperature. Four randomly selected fields were captured using a fluorescence microscope (Nikon company, Tokyo) to calculate the number of cells that had successful invaded and transmigrated the Matrigel.

### CCK-8 assay

Cell proliferation was analyzed by CCK-8 assay (Beyotime Institute of Biotechnology, Beijing) according to the manufacturers’ instructions. Cells were seeded at 5 × 10^3^ cells/well in 96-well plates and cultured for overnight at 37 °C. 10 µl CCK-8 solution was added to each well at different time points. After 4 h of incubation, absorbance was determined at 450 nm using a plate reader (Molecular Devices, LLC, Sunnyvale, CA).

### Apoptosis analysis

Apoptotic cells were analyzed using an Annexin V-FITC/PI staining method. Transfected LACC cells were treated with cisplatin (8 μM) for 2 h. After 24 h cultivation, these cells were stained using the Annexin V-FITC/PI Apoptosis Detection kit according to the manufacturers’ instructions. The percentage of apoptotic cells was measured by flow cytometry (BD Biosciences, Franklin Lakes, NJ).

### Luciferase assay

The luciferase reporter plasmid encoding BRMS1L the three prime untranslated region (3′-UTR) or empty vector, Wnt luciferase activity TOPflash or control FOPflash plasmid were transfected into LACC cells, respectively. Luciferase activities were measured at 48-h post-transfection using the Dual-Luciferase Reporter Assay System (Promega, Madison, WI) according to the manufacturer’s protocol. Renilla luciferase activity was normalized against firefly luciferase activity and presented as percent inhibition. All transfection experiments were performed in triplicate and repeated at least 3 times.

### Western blot

The total protein of the cells was harvested with radioimmunoprecipitation assay buffer (RIPA) containing 1% protease inhibitor (Sigma, St Louis, MO). For each sample, protein (30–50 μg) was separated using 10% sodium dodecyl sulfate polyacrylamide gel electrophoresis (SDS–PAGE) and then transferred into nitrocellulose membranes (BioRad, Hercules, CA). Afterwards, the membrane was blocked with 5% nonfat milk and 0.1% Tween 20 in tris-buffered saline and immunoblotted overnight using β-catenin, BRMS1L, E-cadherin, N-cadherin or glyceraldehyde 3-phosphate dehydrogenase (GAPDH) primary antibodies at 4 °C with gentle shaking. After that, a fluorochrome labelled secondary antibody (Alexa Fluor 800, LI-COR, Lincoln, NE) was used to identify the primary antibodies. Immunoreactivity was detected with the Odyssey fluorescent scanning system (LI-COR) and analyzed by Image Studio software. GAPDH was used as a loading control.

### Immunohistochemical staining

The LACC and control specimens were embedded in paraffin after fixation with 4% paraformaldehyde. 4-μm thick sections for immunohistochemical analysis were prepared, and the sections were deparaffinized and quenched in H_2_O_2_ to block endogenous peroxidase. After rinsed in PBS, the sections were subsequently incubated with antibody against BRMS1L (Abcam, Cambridge, MA) overnight. All images were taken using a microscope (Nikon, Tokyo).

### Statistical analysis

Statistical significance was determined by two-tailed Student’s t-test. Results were expressed as the mean ± standard deviation (SD). The differences were considered to be statistically significant at p < 0.05.

## Results

### Upregulation of miR-93-5p in samples and plasma of LACC patients

In this study, five patients clinically diagnosed with lacrimal gland adenoid cystic carcinoma were enrolled. A summary of clinical features of the patients is listed in Table 1. Briefly, the mean age of the patients was 51 years, with the range between 29 and 81 years. The mean duration of symptoms was around 11 months. Surgery procedures in all cases were attempted to excise tumor tissues completely. Up till now, no patient has experienced a recurrence, with an average follow-up period of 24 months. However, only one patient of the five cases had a follow-up period of more than 4 years (49 months).

**Fig. 1:**
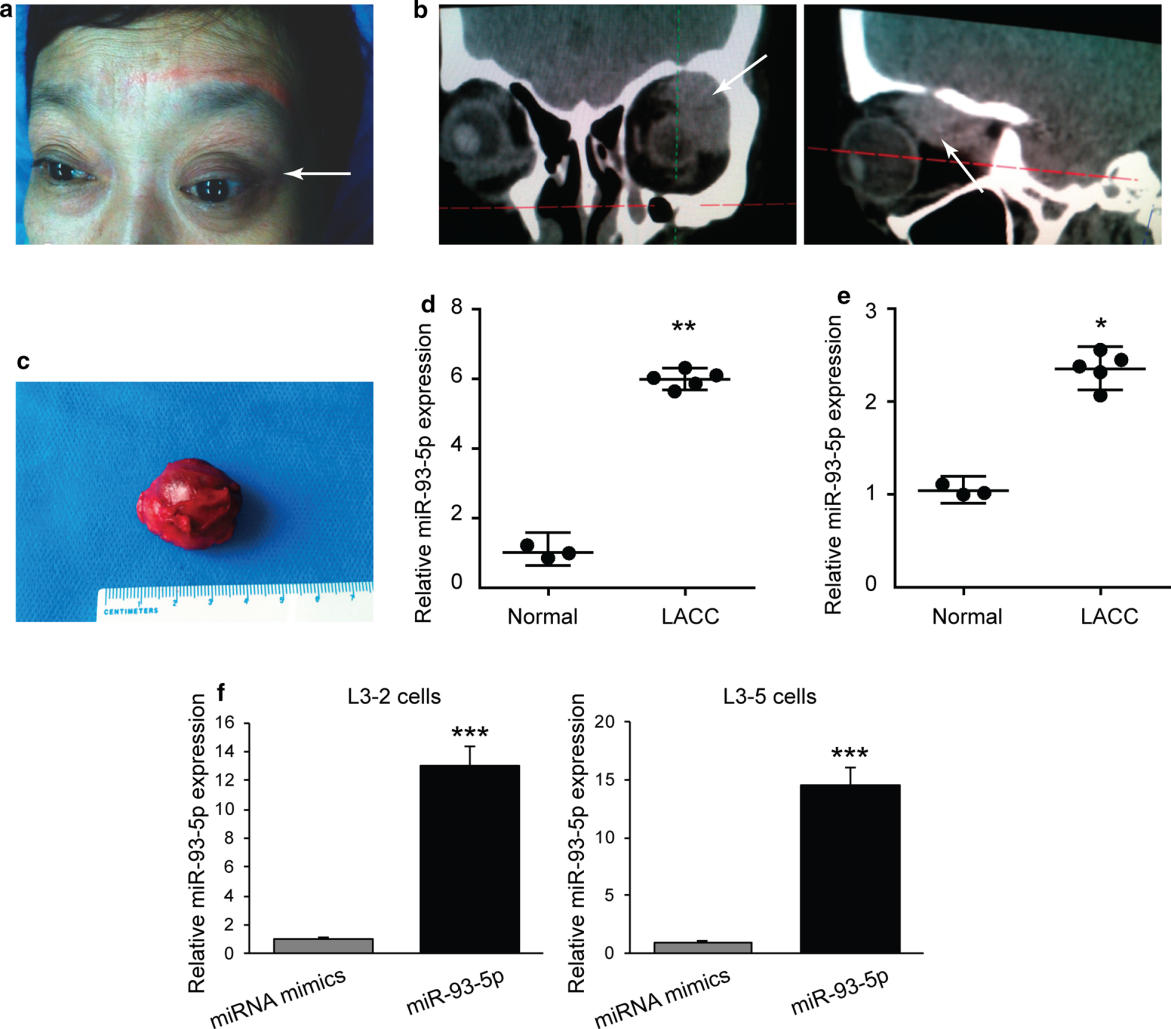
Upregulation of miR-93-5p in samples and plasma of LACC patients. **a** The patient with LACC on right eye. **b** The radiological data showed the proptosis on the right eye with partial destruction of the bone. **c** The LACC tissue obtained from the patient during surgery. The size and the shape of the tumor were shown. **d** The relative expression levels of miR-93-5p in LACC compared to normal tissues. **p < 0.01. **e** The relative expression levels of miR-93-5p in the plasma of LACC patients compared to healthy participants *p < 0.05. **f** LACC cells (L3–2 and L3–5) were isolated from LACC tissues. These cells were then transfected with miRNA mimics or miR-93-5p. The miR-93-5p expression was examined by qRT-PCR after 48 h. **p < 0.001 compared with miRNA mimics cells

Eyelid mass and proptosis of LACC patient were presented (Fig. 1a). The radiologic imaging revealed round, poorly demarcated mass (Fig. 1b), and the removed tumor tissues were round in shape with vascular support (Fig. 1c). The diameters of the tumors ranged from 1.2 to 3.5 cm, with the mean of 2.6 cm. Since miR-93-5p has been implicated in the onset and development of multiple types of cancers [1, 27–29], we first sought to determine whether the expression levels of miR-93-5p were regulated in the tumor tissues of LACC. Using the qRT-PCR technique, we found that compared to normal tissues, there was a 3.2-fold increase in the expression levels of miR-93-5p in the tumor tissues of LACC, which was statistically significant by *t*-test (p < 0.01) (Fig. 1d). We next examined the circulating levels of miR-93-5p in lacrimal gland ACC patients. We found that the levels of miR-93-5p were also significantly upregulated in the plasma of LACC patients, compared to those in the plasma of healthy individual (p < 0.05 by t-test) (Fig. 1e). To investigate whether miR-93-5p affects LACC cell tumorigenesis, we established two LACC cells (L3–2 and L3–5) from fresh LACC tissues and increased miR-93-5p expression by transfection (Fig. 1f).

### Promotive effects of miR-93-5p in LACC cell tumorigenesis

**Fig. 2:**
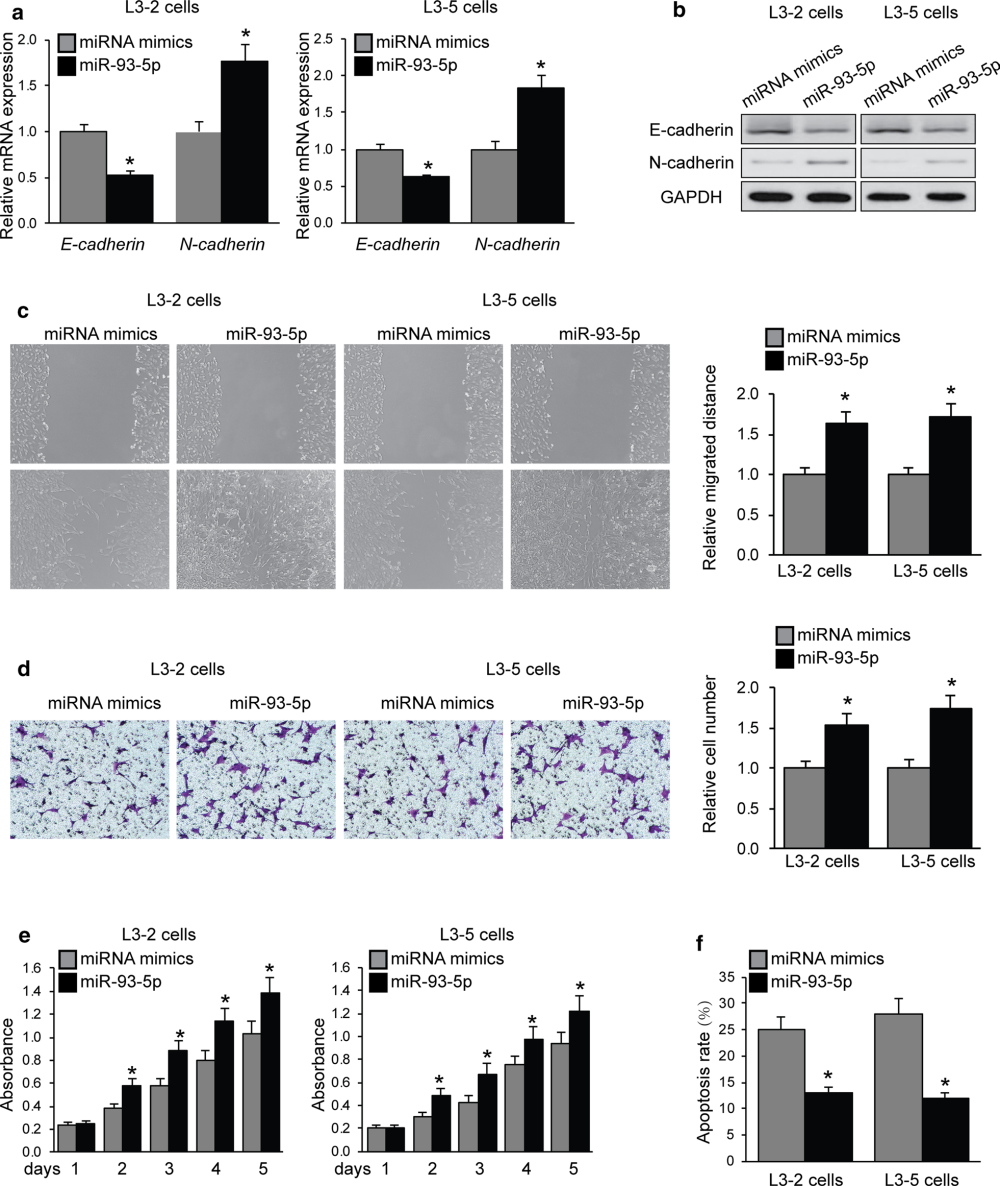
Promotive effects of miR-93-5p in LACC cell migration, invasion and proliferation. L3–2 and L3–5 cells were transfected with miR-93-5p or miRNA mimics. **a** mRNA and **b** protein expression of E-cadherin and N-cadherin was examined by qRT-PCR and western blot. **c** Wounded healing and **d** transwell assays were performed. Representative images (left) and quantification (right) of cell migration and invasion were shown. **e** Cell proliferation was analyzed by CCK-8 assays. **f** Cell apoptosis was examined by Flow cytometry. *p < 0.05 compared with miRNA mimics cells

In order to explore the involvement of miR-93-5p in cell migration and invasion, L3–2 and L3–5 cells with miR-93-5p overexpression were subjected to qRT-PCR and western blot for epithelial–mesenchymal transition (EMT) markers (E-cadherin and N-cadherin) levels. As shown in Fig. 2a, b, significant downregulation of E-cadherin and upregulation of N-cadherin were observed when LACC cells were transfected with miR-93-5p. Ability of LACC cell migration and invasion was examined by wounded healing and transwell assays. As shown in Fig. 2c, d, significant promotion of cell migration and invasion was seen in L3–2 and L3–5 cells with overexpression of miR-93-5p. To analyze the effect of miR-93-5p on LACC cell proliferation, CCK-8 assays were performed when overexpression of miR-93-5p in L3–2 and L3–5 cells. miR-93-5p significantly promoted LACC proliferation (Fig. 2e). These cells were then treated with cisplatin (8 μM) for 1 h, cell apoptosis was analyzed by Flow Cytometry after 24 h. As shown in Fig. 2f, miR-93-5p protected LACC cells from cisplatin-induced apoptosis.

### BRMS1L targeted by miR-93-5p

**Fig. 3:**
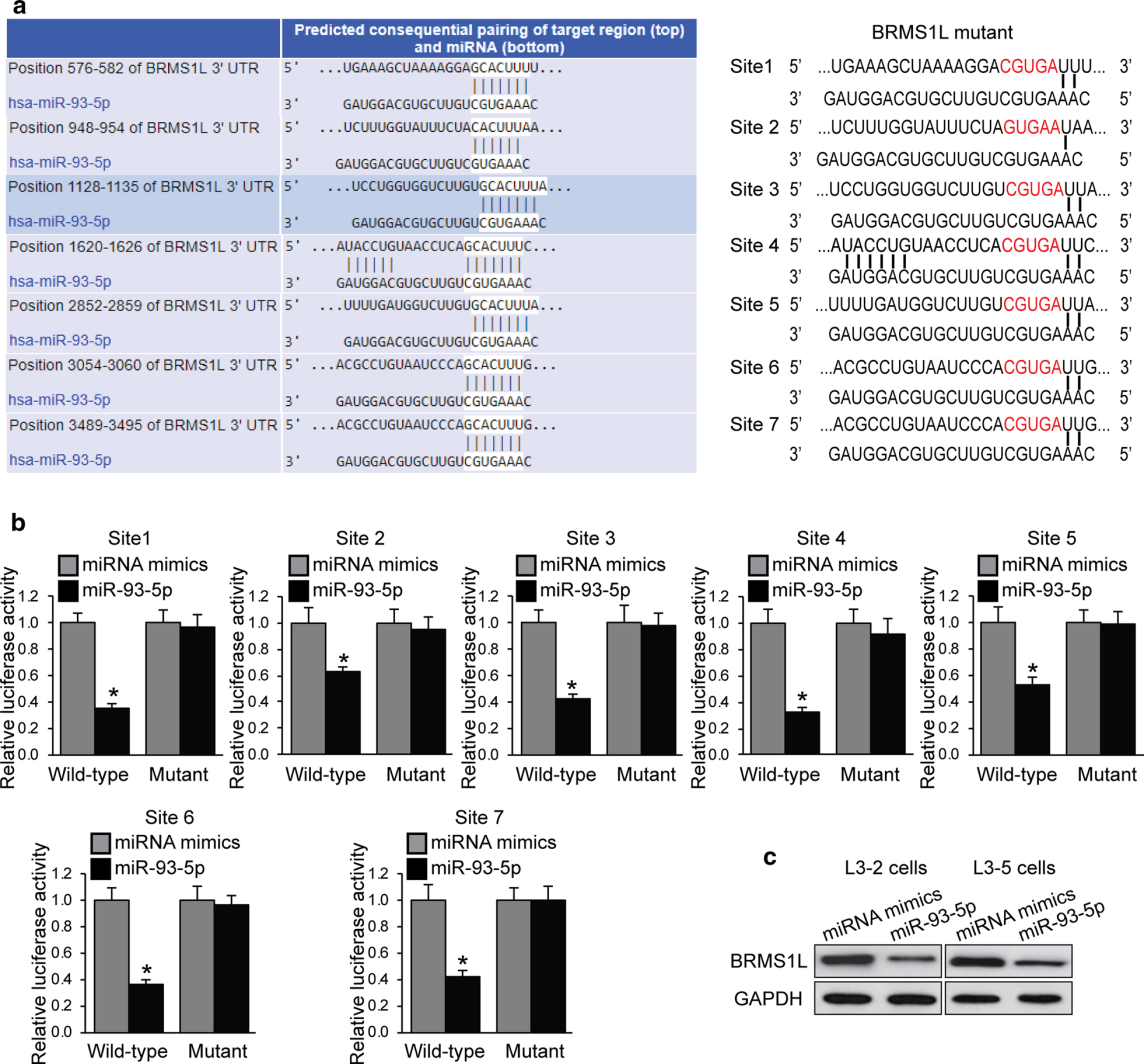
BRMS1L targeted by miR-93-5p. **a** Schematic graph of the 3′UTR of BRMS1L predicted binding sites with miR-93-5p and their mutant. **b** Quantitative analysis of the luciferase activity in 293T cells of reporter vectors containing the BRMS1L 3′UTR of wild type or mutant and miR-93-5p transfection. *P < 0.05 compared with miRNA mimics cells. **c** BRMS1L expression in L3–2 and L3–5 cells by western blot under miRNA mimics or miR-93-5p transfection

To investigate how miR-93-5p affects LACC cell EMT and tumorigenesis, bioinformatic online database (TargetScan) was used to predict putative target of miR-93-5p. Seven binding sites in 3′UTR region of BRMS1L with miR-93-5p were predicted (Fig. 3a). BRMS1L plays an important role in cancer cell metastasis regulation [30, 31]. We then examined whether miR-93-5p targets BRMS1L. We constructed luciferase plasmids containing 3′UTR sequences of BRMS1L with each predicted target site of miR-93-5p and their mutant. Luciferase reporter assays were performed and the results revealed that miR-93-5p significantly suppresses luciferase activity of each wild-type (Fig. 3b). Furthermore, western blotting results further confirmed that miR-93-5p could inhibit BRMS1L protein expression (Fig. 3c). These results indicated that miR-93-5p targets BRMS1L.

### Negative correlation between miR-93-5p and BRMS1L

**Fig. 4:**
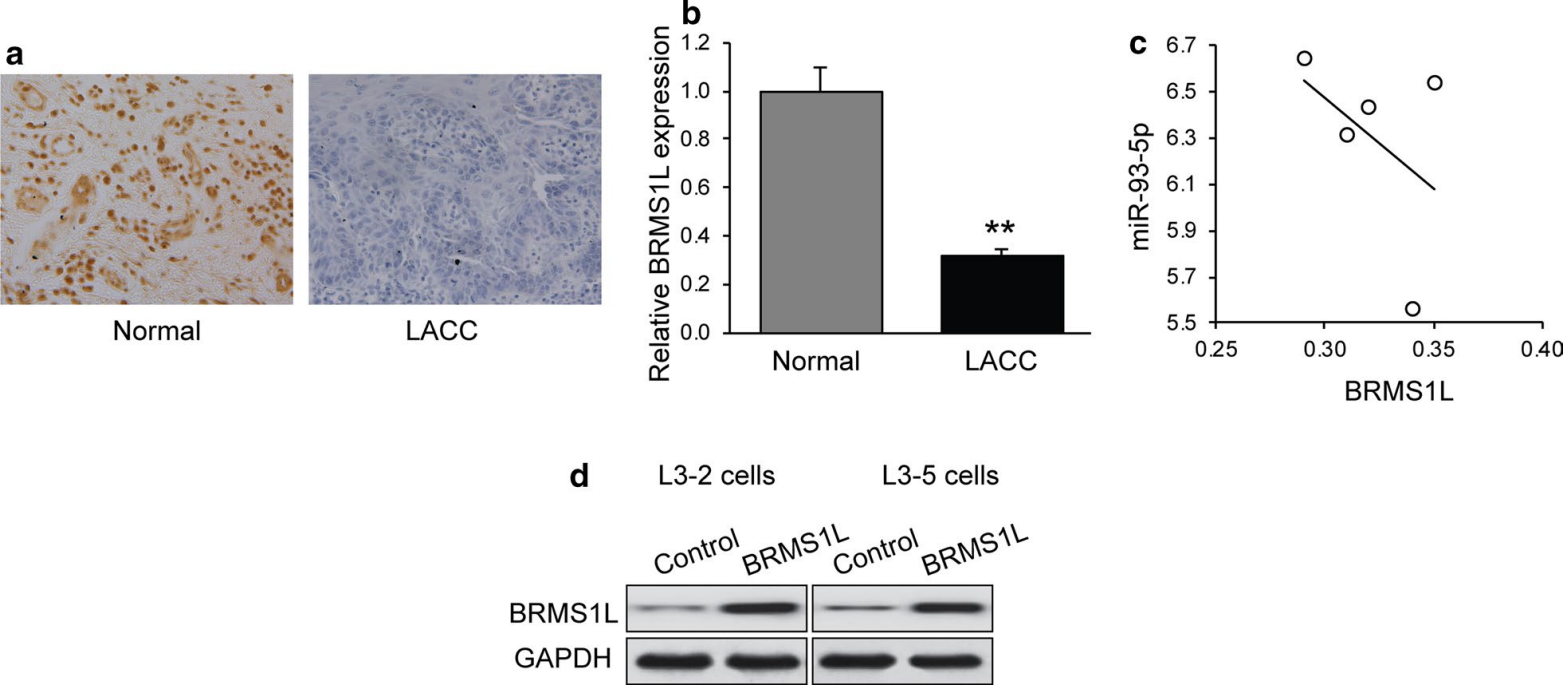
Negative correlation between miR-93-5p and BRMS1L. **a** Immunohistochemical staining of BRMS1L in LACC and normal tissue. **b** The relative expression levels of *BRMS1L* mRNA in LACC compared to normal tissues. **p < 0.01. **c** Correlation analysis of *BRMS1L* and miR-93-5p in LACC tissues (r = 0.438), (p < 0.05). **d** BRMS1L protein expression in L3–2 and L3–5 cells by western blot under BRMS1L plasmid or control vector transfection

To examine the expression levels of BRMS1L in LACC, we performed immunohistochemical staining in LACC tissues for BRMS1L. Compared to normal tissues, the BRMS1L staining in LACC tissues was weaker than normal control (Fig. 4a). Next, qRT-PCR was performed for BRMS1L mRNA expression, as shown in Fig. 4b, where expression of BRMS1L was significantly downregulated in LACC tissues compared with normal tissues. The Pearson’s correlation analysis revealed a reverse correlation between miR-93-5p and the expression of BRMS1L (r = 0.438, p < 0.05) (Fig. 4c). To evaluate the role of BRMS1L in LACC cell migration and invasion, we increased BRMS1L expression in both L3–2 and L3–5 cells (Fig. 4d).

### Inhibit effects of BRMS1L in LACC cell tumorigenesis

**Fig. 5:**
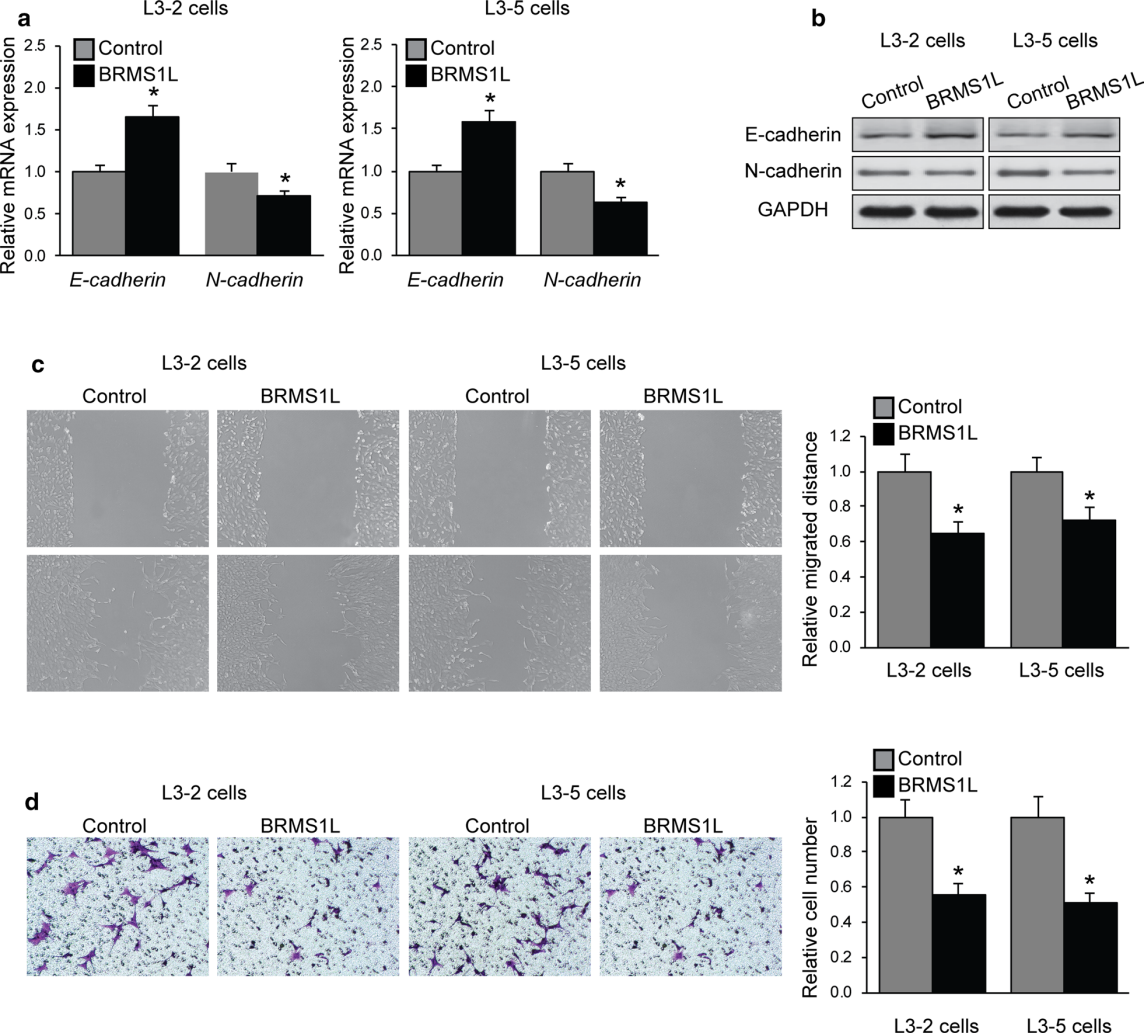
Inhibit effects of BRMS1L in LACC cell migration and invasion. L3–2 and L3–5 cells were transfected with BRMS1L plasmid or control vector. **a** mRNA and **b** protein expression of E-cadherin and N-cadherin was examined by qRT-PCR and western blot. **c** Wounded healing and **d** transwell assays were performed. Representative images (left) and quantification (right) of cell migration and invasion were shown. *p < 0.05 compared with control vector cells

In order to investigate whether BRMS1L determine migration and invasion ability of LACC cells, L3–2 and L3–5 cells with BRMS1L overexpression were subjected to qRT-PCR and western blot for E-cadherin and N-cadherin levels. As shown in Fig. 5a, b, significant upregulation of E-cadherin and downregulation of N-cadherin were found when LACC cells were transfected with BRMS1L. Ability of LACC cell migration and invasion was examined by wounded healing and transwell assays. As shown in Fig. 5c, d, significant inhibition of cell tumorigenesis was observed in L3–2 and L3–5 cells with overexpression of BRMS1L.

### miR-93-5p regulates BRMS1L-mediated Wnt signaling

**Fig. 6:**
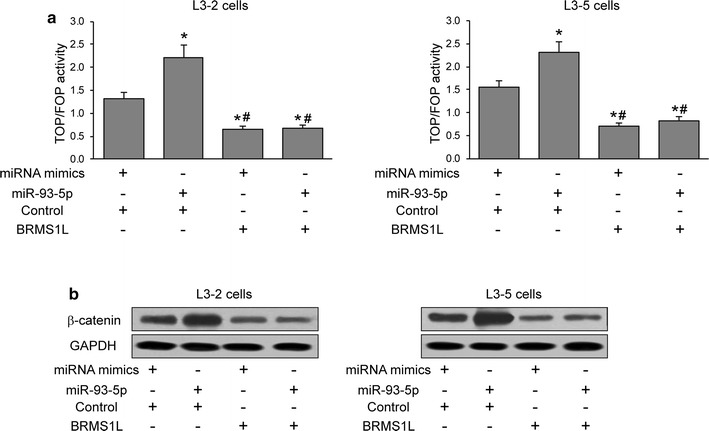
miR-93-5p regulates BRMS1L-mediated Wnt signaling. **a** TOP/FOP luciferase activity in L3–2 and L3–5 cells under miRNA mimics + control vector, miR-93-5p + control vector, miRNA mimics + BRMS1L and miR-93-5p + BRMS1L treatment. **b** Western blot of β-catenin expression in L3–2 and L3–5 cells under miRNA mimics + control vector, miR-93-5p + control vector, miRNA mimics + BRMS1L and miR-93-5p + BRMS1L treatment. * and *# revealed p < 0.05

It has been reported that BRMS1L play a role in regulating Wnt signaling [31]. To examine whether miR-93-5p affects Wnt signaling, TOPflash and FOPflash luciferase plasmids were introduced into L3–2 and L3–5 cells for 48 h, then miR-93-5p or miRNA mimics were transfected into these cells for luciferase activity testing. As shown in Fig. 6, the results revealed that miR-93-5p significantly enhanced TOP/FOP luciferase activity. Upregulation of BRMS1L reduced TOP/FOP luciferase activity while further overexpression of miR-93-5p could not rescue Wnt signaling activity (Fig. 6a). Western blot results further confirmed miR-93-5p failed to rescue β-catenin expression affected by BRMS1L overexpression, indicating that miR-93-5p regulates Wnt signaling through BRMS1L.

## Discussion

The LACC patients were enrolled in the study with the mean age of 51 years, but with a broad range between 29 and 81 years. Based on reported cases, the prognosis of lacrimal gland ACC is much worse in adults compared to that of children and adolescents [12]. Tellado et al. estimated much shorter median survival time of 5 years in ACC for patients of all ages compared to the survival rate of 58% at 15 years [13]. The reported in the literature mean duration of symptoms was around 11 months, but it differs among studies (3–24 months) with an average follow-up period of 24 months (12–49 months). LACC more often affected left side. Treatment protocol for all cases was similar and included tumor resection with no recurrence in any of the patients.

According to the literature, miR-93-5p has been implicated in the development and progression of multiple types of cancers such as gastric cancer [27, 28], breast cancer [29], ovarian cancer [20], colorectal, pancreatic, lung cancer and hepatocellular carcinoma [17]. In our study, the expression level of miR-93-5p was 3.2-fold increased in the tumor tissues of LACC (p < 0.01) as well as significantly upregulated in the plasma of LACC patients, compared to healthy controls (p < 0.05). Our results are consistent with the literature as Fang et al. [18] also reported elevated expression profile of miR-93 in tumor growth and angiogenesis. They found that miR-93 increased cell survival, promoted sphere formation and augmented tumor growth. Therefore, inhibition of miR-93 function may be a credential approach to suppress angiogenesis and tumor growth [18]. Similarly, miR-93 was found to be overexpressed in endometrial carcinoma tissues compared to normal endometrial tissues [17]. Contrariwise, Chen et al. 215 reported decreased levels of miR-93-5p in EOC and borderline tumors compared to normal ovarian tissues as well as in metastatic omentum compared to relative primary ovarian carcinomas [20]. Thus, Chen et al. demonstrated for the first time that miR-93-5p can be a potential suppressor of ovarian cellular proliferation [20].

Recurrence and metastasis are the major causes of death in patients with many types of cancer such as endometrial carcinoma [32]. Tumor invasion and metastasis are closely related to EMT [33]. EMT is an intricate process with the loss of polarity of cancer cells and cell phenotype alteration from epithelial to mesenchymal structure, resulting in an upregulated angiogenesis, enhanced cell motility, and extracellular matrix invasion [34]. MiRNAs are known to regulate diverse basic cellular functions such as proliferation, migration, invasion, and the EMT process [35]. Therefore, the LACC cell lines were transfected with miR-93-5p, after which cell migration and invasion ability and the expression of relevant molecules were detected. MiR-93 overexpression promoted cell migration and invasion, and downregulated E-cadherin expression while increasing N-cadherin expression. Therefore, we suggest that miR-93 may promote the process of EMT (cell metastasis) in LACC cells with overexpression of miR-93-5p.

The miR-93 targets include P21, cyclin B1 (CCNB1), ERBB2 [36], AKT serine/threonine Kinase 3 (Akt3), SRY-related HMG-box 4 (SOX4), signal transducer and activator of transcription 3 (STAT3) [37], vascular endothelial growth factor A (VEGFA) [38], and Smad7 [39], suggesting that miR-93 can be a tumor suppressor by activating different signaling pathways. According to Ma et al., mi-R-93-5p promotes gastric cancer metastasis through activating the STAT3 signaling pathway [the miR-93-5p/interferon alpha/beta receptor 1 precursor (IFNAR 1) axis] [27]. Li et al. [28] reports a gastric cancer progression via inactivation of the Hippo signaling pathway. In addition, miR-93-5p inhibits EMT of breast cancer cells via targeting megakaryoblastic leukemia (translocation) 1 (MKL-1) and STAT3 [29]. In turn, Chen et al. reported for the first time that miR-93 promotes endometrial carcinoma cell EMT, migration, and invasion via targeting downregulation of Forkhead box protein A1 (FOXA1), which differs from its anti-oncogene role in ovarian cancer [17]. Fang et al. [18] reports that miR-93 promotes tumor growth and angiogenesis by suppressing integrin-b8. In turn, Fabbri et al. investigated the role of miR-93-5p on the expression levels of vascular endothelial growth factor (VEGF) and interleukin-8 (IL-8) in the neuroblastoma SK-N-AS cell line [19], while Chen et al. for the first time reported that miR-93-5p hampers EOC tumorigenesis and progression by targeting Ras homolog gene family member C (RhoC) [20].

BRMS1 suppresses metastasis of multiple tumor types without blocking tumorigenesis [21]. BRMS1 forms complexes with SIN3, histone deacetylases and selected transcription factors, thus regulating metastasis-associated gene expression [e.g. epidermal growth factor receptor (EGFR), osteopontin (OPN), phosphatidylinositol-4-phosphate 5-kinase 1 alpha (PI4P5K1A) and urokinase-plasminogen activator (PLAU) [21]. BRMS1L plays an important role in cancer cell metastasis regulation [30, 31] such as breast cancer [22], melanoma [23], non-small cell lung [24] and ovarian cancer [16]. BRMS1L can be activated in many different signaling pathways. It suppresses breast cancer metastasis by inducing epigenetic silence of Frizzled homolog 10 (FZD10) [31]. Reduced BRMS1L in breast cancer tissues are associated with metastasis and poor patient survival through epigenetic silencing of FZD10, a receptor for Wnt signalling, through histone deacetylase 1 (HDAC1) recruitment and histone H3K9 deacetylation at the promoter, followed by the inhibition of the aberrant activation of WNT3-FZD10-β-catenin signalling. RNA interference-mediated silencing of BRMS1L expression promotes metastasis of breast cancer xenografts. Thus, BRMS1L provides an epigenetic regulation of Wnt signalling and suppresses a breast cancer metastasis [31].

Therefore, we investigated if miR-93-5p targets BRMS1L. Luciferase reporter assay showed that miR-93-5p directly binds to the 3′UTR of BRMS1L in LACC. Furthermore, miR-93-5p overexpression downregulated expression of BRMSIL in LACC tissues compared with normal tissues. In addition, transfection with BRMS1L resulted in significant inhibition of cell metastasis in LACC cells with overexpression of BRMS1L.

Since in the literature, it was reported that BRMS1L play a role in regulating Wnt signaling [31], we decide to examine this pathway more in depth. In our study, luciferase activity testing showed that miR-93-5p significantly enhanced TOP/FOP luciferase activity. In turn, upregulation of BRMS1L reduced TOP/FOP luciferase activity while further overexpression of miR-93-5p did not recover Wnt signaling activity. Therefore, we suggest that miR-93-5p may promote the process of EMT in LACC cells and regulates Wnt signaling by targeting BRMS1L.

## Conclusions

Our findings report for the first time that miR-93 promotes LACC cell EMT, migration, and invasion via targeting downregulation of BRMS1L through regulation of Wnt signaling pathway, which differs from its anti-oncogene role in ovarian cancer. However, further preclinical studies are necessary to determine the role of the same miRNA in different malignant tumors. The exact mechanisms underlying this pathway may lead to important treatment strategies which can significantly reduce morbidity and mortality in LACC patients.

## Additional file

**Additional file 1: Table S1.** Primers used for qRT-PCR.

## Abbreviations

ACC: adenoid cystic carcinoma
LACC: lacrimal gland adenoid cystic carcinoma
RT: external radiotherapy
miRNAs: microRNAs
EOC: epithelial ovarian carcinoma
BRMS1: breast cancer metastasis suppressor 1
BRMS1L: breast cancer metastasis-suppressor 1 like
qRT-PCR: quantitative reverse transcription polymerase chain reaction
PBS: phosphate buffered saline
DMEM/F-12: Dulbecco’s Modified Eagle Medium: Nutrient Mixture F-12
3′-UTR: three prime untranslated region
RIPA: radioimmunoprecipitation assay buffer
SDS-PAGE: sodium dodecyl sulfate polyacrylamide gel electrophoresis
GAPDH: glyceraldehyde 3-phosphate dehydrogenase
SD: standard deviation
EMT: epithelial–mesenchymal transition
CCNB1: cyclin B1
Akt3: AKT serine/threonine kinase 3
SOX4: SRY-related HMG-box 4
STAT3: signal transducer and activator of transcription 3
VEGFA: vascular endothelial growth factor A
IFNAR 1c: interferon alpha/beta receptor 1 precursor
MKL-1: megakaryoblastic leukemia (translocation) 1
FOXA1: forkhead box protein A1
VEGF: vascular endothelial growth factor
IL-8: interleukin-8
EOC: epithelial ovarian carcinoma
RhoC: Ras homolog gene family member C
FZD10: frizzled homolog 10
HDAC1: histone deacetylase 1
EGFR: epidermal growth factor receptor
OPN: osteopontin
PI4P5K1A: phosphatidylinositol-4-phosphate 5-kinase 1 alpha
PLAU: urokinase-plasminogen activator
