# Supplementary material for: miR-93-5p enhance lacrimal gland adenoid cystic carcinoma cell tumorigenesis by targeting BRMS1L
Source: Cancer Cell Int. 2018 May 9;18:72. doi: 10.1186/s12935-018-0552-9 (PMC5944175; doi:10.1186/s12935-018-0552-9)
Supplement: Supplementary file 1 — Additional file 1: Table S1. Primers used for qRT-PCR. [file 12935_2018_552_MOESM1_ESM.doc]

Additional file 1: Table S1. Primers used for qRT-PCR.

| primers | Sequences (5’-3’) |
| --- | --- |
| miR-93-5p forward | ACACTCCAGCTGGGCAAAGTGCTGTTCGTGC |
| miR-93-5p reverse | CTCAACTGGTGTCGTGGAGTCGGCAATTCAGTTGAGCTACCTGC |
| E-cadherin forward | CCCACCACGTACAAGGGTC |
| E-cadherin reverse | CTGGGGTATTGGGGGCATC |
| N-cadherin forward | CAACTTGCCAGAAAACTCCAGG |
| N-cadherin reverse | ATGAAACCGGGCTATCTGCTC |
| BRMS1L forward | GAGCGGTTGAGTCAGGTGG |
| BRMS1L reverse | CCTTTGTGCGAATCTGCATGT |
| GAPDH forward | GATGCTGGCGCTGAGTACG |
| GAPDH reverse | GCTAAGCAGTTGGTGGTGC |
